# The Role of Supplementation with Natural Compounds in Post-Stroke Patients

**DOI:** 10.3390/ijms22157893

**Published:** 2021-07-23

**Authors:** Natalia Cichon, Joanna Saluk-Bijak, Elzbieta Miller, Leslaw Gorniak, Justyna Redlicka, Marta Niwald, Michal Bijak

**Affiliations:** 1 Biohazard Prevention Centre, Faculty of Biology and Environmental Protection, University of Lodz, Pomorska 141/143, 90-236 Lodz, Poland; leslaw.gorniak@biol.uni.lodz.pl (L.G.); michal.bijak@biol.uni.lodz.pl (M.B.); 2 Department of General Biochemistry, Faculty of Biology and Environmental Protection, University of Lodz, Pomorska 141/143, 90-236 Lodz, Poland; joanna.saluk@biol.uni.lodz.pl; 3 Department of Neurological Rehabilitation, Medical University of Lodz, Milionowa 14, 93-113 Lodz, Poland; elzbieta.dorota.miller@umed.lodz.pl (E.M.); justyna.redlicka@umed.lodz.pl (J.R.); marta.niwald@umed.lodz.pl (M.N.)

**Keywords:** stroke, supplementation, natural compounds, vitamins, flavonoids, macro-elements, antioxidants, anti-inflammatory agents, neuroprotection, neuroplasticity

## Abstract

Malnutrition is a serious problem in post-stroke patients. Importantly, it intensifies with hospitalization, and is related to both somatic and psychological reasons, as well as is associated with the insufficient knowledge of people who accompany the patient. Malnutrition is a negative prognostic factor, leading to a reduction in the quality of life. Moreover, this condition significantly extends hospitalization time, increases the frequency of treatment in intensive care units, and negatively affects the effectiveness of rehabilitation. Obtaining growing data on the therapeutic effectiveness of new compounds of natural origin is possible through the use of pharmacodynamic and analytical methods to assess their therapeutic properties. The proper supply of nutrients, as well as compounds of natural origin, is an important element of post-stroke therapy, due to their strong antioxidant, anti-inflammatory, neuroprotective and neuroplasticity enhancing properties. Taking the above into account, in this review we present the current state of knowledge on the benefits of using selected substances of natural origin in patients after cerebral stroke.

## 1. Introduction

People with neurological diseases, including poststroke patients, are particularly prone to malnutrition. That malnutrition is caused by ailments associated with neurological diseases, such as impaired consciousness and cognitive functions, neurogenic dysphagia, neurogenic vomiting, gastrointestinal dysfunction, motor deficits or depression [1,2,3]. According to ESPEN (European Society for Clinical Nutrition and Metabolism), malnutrition is defined as “a condition resulting from the lack of intake or absorption of nutrients, leading to a change in body composition, physical and intellectual impairment of the body’s physical and intellectual function, and adversely affecting the treatment of the underlying disease” [4]. It results from the lack of absorption or limited supply of nutrients, leading to changes in body composition and impairment of physical and intellectual functions. There are discrepancies in the reports of malnutrition in patients after stroke, some studies report the incidence ranges from 6.1% to even 62% [5,6], while other studies report that the rates range from 8% to 49% [7].

The proper assessment of the nutritional status and its monitoring are important elements of preventive and therapeutic management in neurological diseases. Among the causes of malnutrition are decreased nutrient intake as a result of, e.g., esophageal stricture or obstruction, dysphagia, deterioration of general condition or disability; digestive disorders; increase of catabolic processes as a result of the action of immunological and humoral factors, loss of nutrients, inter alia as a result of dialysis or nephrotic syndrome; pharmacotherapy [8].

**Figure 1:**
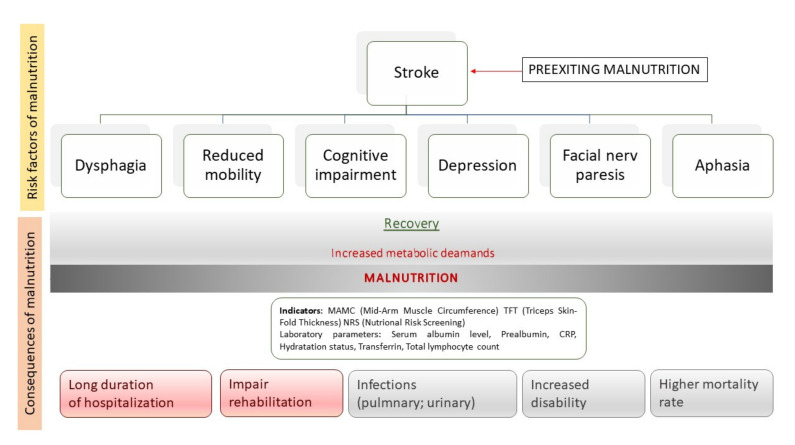
Risk factors and consequences of post-stroke patient’s malnutrition.

Malnutrition is a negative prognostic factor, it significantly increases the risk of pressure ulcers and infections, in particular of the respiratory system, water and electrolyte disturbances, anemia, coagulation disorders, bradycardia, osteoporosis, leading to a reduction in the quality of life (Figure 1). Moreover, this condition significantly extends hospitalization time, increases the frequency of treatment in intensive care units, and negatively affects the effectiveness of rehabilitation. Reducing the concentration of proteins reduces muscle strength as well as impairs immunity [3]. Importantly, in a clinical trial FOOD (Feed Or Ordinary Diet, 4023 participants, aged 71 ± 12.5, follow up period 6 months) it has been shown that malnutrition is associated with increased mortality in patients after stroke (odds ratio (OR) 2.32, 95% confidence interval (CI) 1.78–3.02) [9].

Currently, there is a search for new compounds of plant origin, as well as those contained in fungi, which can be used in the treatment of chronic or incurable diseases. In recent years, research has shown that ingredients of plant and fungal origin, in addition to nutritional values, can prevent civilization disorders, including cerebrovascular disease. Obtaining growing data on the therapeutic effectiveness of these preparations is possible through the use of pharmacodynamic and analytical methods to assess the therapeutic properties of the obtained substances and their effects. The proper supply of nutrients, as well as compounds of natural origin, is an important element of post-stroke therapy. For this reason, this review presents the current state of knowledge about the benefits of supplementation in stroke patients.

## 2. Natural Compounds

Vitamin and mineral deficiencies may be particularly precarious for people with cerebrovascular diseases, in whom appropriate diet therapy may help to correct risk factors and improve the prognosis for the further course of the disease. The effects of non-pharmacological activities in the primary and secondary prevention of cerebrovascular diseases, as well as in neuroprotection, may sometimes equal complicated and expensive medical procedures. Compounds with potential neurorestorative activity include exogenous antioxidants: polyphenols, polyunsaturated fatty acids (PUFAs), vitamins A and C, as well as vitamin D. Vitamins B6 and B12 and folates are also important due to their importance in the metabolism of homocysteine. Additionally, micronutrients such as zinc and selenium have a protective effect on central nervous system functions.

### 2.1. Vitamins

Vitamin D (VitD), a fat-soluble steroidal organic chemical, plays a key role in calcium homeostasis. Moreover, it also promotes cell proliferation, participates in immunomodulation and control of other endocrine systems [10]. Endogenous synthesis of VitD occurs under the influence of sunlight, while the food sources are fish, fortified dairy products and eggs [11]. The vitamin D receptor (VDR) is found in a variety of cells, including the endothelium and vascular smooth muscle. The biological activity of VitD is related to the regulation of genes involved in the control of the cell cycle, apoptosis, membrane transport, oxidative stress, cell adhesion, and matrix homeostasis. The immunomodulating effect is expressed through the upregulation of metalloproteinase inhibitors and anti-inflammatory cytokines, as well as the reduction of the expression of metalloproteinases, proinflammatory cytokines and natriuretic peptides [12]. In recent years, convincing data have been obtained on the link between VitD deficiency and the development of cerebrovascular accidents. Turetsky et al. observed that a higher concentration of 25-hydroxyvitamin D (25 [OH] D) was associated with a lower infract volume, regardless of the results of the NIHSS scale or the etiology of lacunar infarction. Importantly, they showed that a 10 ng/mL reduction in 25 (OH) D concentration was associated with a doubling of the risk of a poor outcome after 90 days [13]. Growing evidences demonstrate that decreased VitD level correlated with stroke severity, infarct volume, and mortality after stroke [14,15,16]. Zhang et al. determined the relationship between the level of VitD and the prognosis and clinical condition of patients after acute stroke. The study included 377 patients (241 men and 136 women), aged 64.9 ± 13.7 years, the follow-up period was 3 months. In patients with arterial hypertension, the level of VitD was not associated with the severity of stroke (according to NIHSS) on admission and with poor functional results (according to the Rankin scale), but such a correlation was shown in the group of patients without hypertension (OD 5.51, 95% CI 95% 1.83–16.60 and OD 4.63, 95% CI 1.53–14.05) [15]. In turn, during a 24-month follow-up of post-stroke patients (216 participants, 133 men and 83 women, aged 65 (IQR 55–76)) Qiu et al. showed that VitD deficiency was associated with an increased risk of stroke reoccurrence (OD 0.77, 95% C 0.70–0.86, *p* < 0.001) and mortality (OR 0.72, 95% CI 0.64–0.80, *p* < 0.001) [16]. Moreover, a retrospective clinical study by Yalbuzdag et al. showed poorer scores for physical and mental post-stroke outcomes in patients with VitD deficiency. The aim of the study was to investigate the relationship between the level of VitD and the functional results of patients after stroke during rehabilitation. The study included 120 patients up to 36 months after the stroke, 64 men and 56 women, whose mean age was 63.38 ± 11.8. [17]. Despite studies showing a relationship between VitD deficiency and the severity of stroke and poor prognosis, a meta-analysis by Khan et al. (involved a total of 277 trials, 24 interventions, 992,129 participants) showed that vitamin D supplementation did not affect the risk of stroke. In contrast, the combination of vitamin D and calcium increased the risk of an ischemic event, which may be related to hypercalcemia-mediated vascular calcifications, hypercoagulability and induction of atherosclerosis (RR 1.17, 95% CI 1.05–1.30) [18]. Nevertheless, due to increasing data on the harmfulness of calcium supplementation, it seems that it is not related to VidD intake, but Ca^2+^ consumption [19,20]. The neuroprotective effects of VitD are not fully understood. Improvement of cerebral blood flow, reduction of blood pressure, and vasodilation by increasing the activity of nitric oxide synthase (NOS) has been proposed as a potential VitD neuroprotective mechanism [17]. Moreover, VitD enhanced the expression of neurotrophic factors (vascular endothelial growth factor—VEGF, stromal cell-derived factor 1α—SDF1α, and insulin-like growth factor 1—IGF-1), thus reducing neuronal degeneration [14,17]. In animal models, VitD prevents blood–brain barrier (BBB) disturbance by inhibiting oxidative stress and regulation of tight-junction protein occludin and claudin-5 expression [21].

B vitamins are directly involved in the proper functioning of the nervous system, including cognitive functions. Vitamin B6 (pyridoxine) affects the production of serotonin, dopamine, aminobutyric acid, increases the efficiency of thought processes, prevents apathy and insomnia [22]. Vitamin B12 (cobalamin) is involved in the formation of the myelin sheath [23]. Folic acid is a coenzyme in monocarbon radical transfer reactions [24]. Folate and vitamins B6 and B12 are directly involved as coenzymes in the methylation reactions of homocysteine, an amino acid whose high level intensified atherosclerotic processes and increased the risk of stroke.

Meta-analysis published in Cochrane Library demonstrated that supplementation of vitamin B12, folic acid, and vitamin B6 reduced plasma total homocysteine (tHcy) level and decreased the stroke risk by about 10% (RR 0.90, 95% CI 0.82–0.99) compared with placebo [25]. In turn, in meta-analysis involving 82,723 participants, Zhao et al. observed that folic acid supplementation decreased the risk of stroke overall by 11% (RR 0.89, 95% CI 0.84–0.96) [26]. What is more, in two clinical trials it was observed that B vitamins promoted the improvement of cognitive function and reduction of dementia. Earlier clinical trials did not confirm the effectiveness of B vitamins in stroke prevention, but Spence et al. presented a potential cause of this. The form of vitamin B12, cyanocobalamin, impairs kidney function and increases the risk of cardio- and cerebro-vascular events in patients with renal dysfunction. Thus, when considering renal function, they demonstrated that in patients with renal dysfunction, vitamin B supplementation did not reduce the risk of stroke (RR 1.04, 95% CI 0.84–1.27), but in patients without impaired renal function, vitamin B supplementation showed a preventive effectiveness (RR 0.78, 95% CI 0.67–0.90; *p* = 0.03) [27].

Carotenoids, lipophilic antioxidant vitamins, are responsible for the yellow-red color of fruits and vegetables, which play an important role in maintaining a good health state. Carotenoids can be divided into: carotenes (α-carotene, β-carotene, lycopene, β-Apo-8′-carotenal), torulene, isorenieratene and their oxygen derivatives xanthophylls [28]. Literature data indicate that plasma carotenoids levels are reduced after stroke and are associated with infarct volume and the level of neurological deficits [29,30]. In a cohort study involving 165 post-stroke patients (age >65 years, 50.3% woman), it was shown that higher plasma carotenoids levels were associated with a lower risk of death from stroke (HR 0.29, 95% CI 0.12–0.71) [31]. Stroke-prone spontaneously hypertensive rat studies showed that β-carotene decreased fibroblast growth factor-1 (FGF1)-mediated gliosis of astrocytes by increasing the expression of genes related to cholesterol regulation: Abcg2, Abca1, Hmgcr, and Apoe [32]. Importantly, observational studies on the relationship between β-carotene levels and the risk of stroke supplied inconsistent results. On the one hand, Hak et al. found a reverse relationship between the plasma level of carotenoids (β-carotene, α-carotene, lycopene) and the risk of stroke (OR 0.62, 95% CI 0.38–1.01, OR 0.59, 95% CI 0.36–0.98, OR 0.61, 95% CI 0.37–1.00, respectively), confirmed in a population study by Sansawa et al. [33,34]. However, on the other hand, in Alpha-Tocopherol, Beta-Carotene Cancer Prevention Study (ATBC Study) it was also shown that β-carotene supplementation did not reduce the risk of stroke and its subtypes (RR 0.97, 95% CI 0.86–1.09). The ATBC Study included 28,519 male cigarette smokers (aged 50–69), which were supplemented with 20 mg of β-carotene [35].

Vitamin C is the most famous and popular vitamin with a multidirectional effect on the human body, it is involved in many important reactions, stimulating various biochemical processes in the body. The unique structure of ascorbic acid, which contains two adjacent groups, hydroxyl and carbonyl, makes this molecule an excellent hydrogen or electron donor. Vitamin C belongs to the group of water phase antioxidants that inhibit the initiation of free radical chain reactions. Vitamin C neutralizes short-lived hydroxyl, oxyalcohol, superoxide, and nitrogen radicals through hydrogen donation, creating stable and nonreactive ascorbic radicals. These radicals are regenerated to form ascorbic acid with the participation of glutathione [36]. Similarly, as with carotenoids, the evidence of vitamin C’s protective effects on cerebrovascular disease is inconclusive. During 20-years follow-up (880 men and 1241 women, >40 years old), Yokoyama et al. found that the higher serum ascorbic acid level was related to lower stroke risk, both ischemic and hemorrhagic. Additional consideration of total cholesterol level, blood pressure, BMI, physical activity, alcohol consumption, smoking, atrial fibrillation, and antihypertensive drugs did not significantly reduce these relationships [37]. Similar data were obtained from the prospective EPIC-Norfolk study (20,649 participants, aged 40–79, both genders, average follow-up 9.5 years) which showed that vitamin C level in the top quartiles reduced the risk of stroke by 42% (RR 0.58, 95% CI 0.43–0.78) compared to the lowest quartile, regardless of sex, age, BMI, smoking, blood pressure, prevalent myocardial infarction and diabetes, physical activity, and social class [38]. The meta-analysis conducted by Al-Khudairy et al. was aimed at assessing the effect of vitamin C supplementation in the primary prevention of vascular disease, which included 14,641 participants. It has been shown that ascorbic acid compared with the placebo group decreased the risk of total stroke (HR 0.89, 95% CI 0.74–0.07) [39]. However, the PHS II clinical trial (14,641 male, aged ≥ 50 years, including 5.1% men with prevalent CVD at randomization) showed that supplementation with vitamin C (at a dose of 500 mg/day) had no effect on both the risk of an ischemic event (HR 0.89, 95% CI, 0.74–1.07; *p* = 0.21) and cardiovascular mortality (HR 1.02, 95% CI 0.85–1.21; *p* = 0.86) [40]. In a meta-analysis involving 188,209 participants, Ye et al. obtained similar results. They observed that supplementation with ascorbic acid did not affect the risk of stroke (RR 0.99, 95% CI 0.93–1.05) [41]. Oxidized vitamin C—dehydroascorbic acid—is capable of penetrating the BBB through the GLUT1 receptor [42]. In preclinical I/R brain injury studies in primates, rats and mice, parenterally administered dehydroascorbic acid, showed that dehydroascorbic acid dose-dependently reduced infract volume, mortality, edema, and neurological disorders. Moreover, in dehydroascorbic acid-treated animals, neurological outcomes as well as blood flow were improved [42,43,44,45].

A recently published meta-analysis of vitamin and mineral supplementation on the outcome of cardiovascular disease and all-cause mortality showed that widespread use of B vitamins as well as folic acid reduces the risk of stroke (RR 0.90, *p* > 0.05 and RR 0.80, *p* > 0.01, respectively). In contrast, supplementation with vitamin D (RR 0.99, 95% CI 0.95–1.03, *p* = 0.58, high-quality evidence), multivitamin supplements, calcium and vitamin C was not associated with a decreased in the risk of cardiovascular disease or a reduction in all-cause mortality. It should be noted, however, that the use of niacin (at a dose of 1–3 g/day) with statins increased all-cause mortality by 10%. The biggest limitation of this work was the fact that the analysis of the results of cohort studies and the fixed effects model were not included, but only the random effects model [46]. Thus, in order to unequivocally determine the effect of vitamin supplementation in the primary prevention of thigh, further multi-center, long-term studies with standardization of the doses used are required.

### 2.2. Flavonoids

The most numerous group of polyphenols are flavonoids, which include flavanones, flavanols, flavones, isoflavones, flavonols, and anthocyanins, as well as biflavonoids, flavonolignans, prenylflavonoids, flavonoid glycosides, chalcones and proanthocyanins [47]. Several observational studies have shown that flavonoid-rich food consumption has a positive effect on improving cognitive function, regardless of age and medical history [48,49,50,51]. Pure flavonoids such as lutein, quercetin, hesperetin, and genistein have the ability to reduce the expression of proinflammatory markers, including tumor necrosis factor alpha—TNF-α, interleukin 1β—IL-1β, and interleukin 6—IL-6 [12]. Zhang et al. investigated the effect of a flavonoid-rich extract (FRE) obtained from *Rosa laevigata* Michx fruit (RLMF) on ischemia/reperfusion (I/R) injury in rats. They demonstrated the neuroprotective effect of FRE (in a dose of 50–200 mg/kg) by reducing neuronal apoptosis and scavenging free radicals, as well as inhibiting neuroinflammation. FRE caused a reduction of proinflammatory biomarker expression (IL-1β, IL-6, IL-4, TNF-α, inducible nitric oxide synthase—iNOS, nuclear factor kappa-light-chain-enhancer of activated B cells NFκB, matrix metalloproteinase-9—MMP-9, and cyclooxygenase-2—COX-2), as well as decrease in the level of protein kinase RNA-like kinase endoplasmic reticulum (p-ERK), N-terminal c-jun kinase (p-JNK), and members of mitogen-activated protein kinase (MAPK) pathway [52]. The antioxidant effect of flavonoids is based on scavenging free radicals, chelating metals, and silencing pro-oxidative enzymes (xanthine oxidase, protein kinase C—PKC, nicotinamide adenine dinucleotide phosphate (NADPH) oxidase, COX and lipoxygenase) [12]. The molecular neuroprotective mechanism of these compounds is often associated with the activation of the cAMP response element-binding protein (CREB)/brain-derived neurotrophic factor (BDNF)/tropomyosin-related kinase B receptor (TrkB)/phosphoinositide 3-kinase (PI3K)/protein kinase B (Akt) and/or ERK 1/2 pathways, therefore even small doses of these compounds can cause a synergistic effect [47]. One of the most abundant naturally occurring flavonoids is quercetin, contained, among others, in cabbage, spinach, blackcurrant, blueberry, thus in plants particularly recommended in the MIND diet, but it is also the active ingredient of many supplements. The well-documented effect of quercetin is associated with its anti-oxidant, -inflammatory, -hypertensive, -platelet, -atherosclerotic, -obesity, -hypercholesterolemic, -cancer, and -allergic properties [53]. In vitro studies have documented that quercetin has neuroprotective effects also by inhibiting cellular toxicity [54]. In randomized controlled clinical trial, Loke et al. observed, that quercetin enhanced endothelial function by reducing endothelin 1 levels and increasing NO levels [55]. In addition, Egert et al. assessed the effect of quercetin supplementation (150 mg/day for 6 weeks, 93 obese or overweight participants aged 25–65 years) on cardiovascular disease risk factors: blood pressure, body composition, metabolism lipids, markers of inflammation and oxidative stress. They showed that this flavonol reduced systolic blood pressure both in the general population as well as in the hypertensive subgroup and the younger adult subgroup (by 2.6 mmHg, *p* < 0.01; by 2.9 mmHg, *p* < 0.01; o 3.7 mmHg, *p* < 0.001 respectively) compared to placebo. In the quercetin group, serum HDL levels were lowered, while TAG, total cholesterol, LDL-HDL, and TAG-HDL, as well as inflammatory parameters and nutritional status, remained unchanged. Importantly, this study did not find any side effects of quercetin based on laboratory studies [56]. N However, in two other clinical trials, in which healthy volunteers were enrolled, no effect of quercetin on blood pressure and endothelial function was observed (Bondonno et al. at a dose of 0–400 mg/day; Downer et al. 160 mg/day) [57,58]. However, a meta-analysis by Serban et al. (7 clinical trials, 587 participants) demonstrated that quercetin reduced both systolic blood pressure (WMD) −3.04 mmHg, 95% CI −5.75–−0.33, *p* < 0.05) and diastolic blood pressure (WMD −2.63 mmHg, 95% CI −3.26–−2.01, *p* < 0.001). However, when considering the quercetin dose, only a dose >500 mg/day has been shown to have antihypertensive effects on both systolic and diastolic pressure [59].

In turn, epigallocatechin gallate (EGCG), the source of which is green tea, is a powerful antioxidant with anti-inflammatory and suppressive properties, belonging to the flavon-3ol. Neuroprotective effect of EGCG in stroke has been well-known [60,61,62]. The neurorestorative properties of EGCG are related to the activation of CREB/BDNF/TrkB-PI3K/Akt signaling, as demonstrated by the noted increases in Akt, phospho-Akt, mTORc1 and phospho-glycogen synthase kinase 3 (pGSK3b), as well as growth in *BDNF* and *TrkB* expression [63]. Nan et al. found that EGCG treatment decreased neurological deficits, reduced the level of brain injury and oxidative stress biomarkers, inhibited neuronal apoptosis, and promoted neuron survival in a rat model with I/R [61].

Baicalin is 7-O-glucuronide of baicalein, which occurs naturally in *Scutellaria*, while in Chinese medicine as a dietary supplement it is a popular antiviral, antibacterial, anti-inflammatory, antiapoptotic, anticoagulant and antioxidative substance [64]. In in vivo study has been observed, that treatment of baicalin enhanced cognitive, behavioral and motor functions, improved neurological deficit, and decreased the infarct volume [65,66,67,68,69]. Intravenous injection of baicalin (in doses of 100 and 200 mg/kg) to rats 24 h after I/R injury resulted in a statistically significant reduction in neurological scores compared to the control group (1.3 ± 0.5, *p* < 0.05 and 1.2 ± 0.4, *p* < 0.01 vs. 2.0 ± 0.4, respectively), while at the dose of 50 mg/kg no differences were noted. In contrast, the infract volume was reduced in all baicalin-treated animals compared to MCAO animals: at a dose of 50 mg/kg to 27% (*p* < 0.05), 100 mg/kg to 17% (*p* < 0.01) and 200 mg/kg to 12% (*p* < 0.01). [69]. Neurorestorative effects of baicalin were associated with the modulation of mitochondrial function and the suppression of CaMKII phosphorylation. Furthermore, it has been noted, that supplementation of baicalin in the dose of 50 and 100 mg/kg for 7 days dose-dependently enhanced synaptic plasticity in the hippocampus of MCAO [67]. Another proposed neuroprotective mechanism of baicalin is the modulation of the toll-like receptor 2 and 4 (TLR 2/4) pathways. It has been observed that this flavonoid decreased the serum levels of TNF-α and IL-1β, TLR 2/4 and NF-κB expression, as well as both expression and activity of iNOS and COX-2 [67,69,70]. Reduction of neurogenesis disorder in the hippocampus and cognitive disorder in γ-ray radiation-exposed mice after baicalin treatment has been observed. It was caused by suppression of oxidative stress and activation of BDNF/CREB pathway [70]. Currently, the beneficial effects of baicalin on I/R injuries, well documented in in vitro and animal models studies, have not been translated into clinical trials. However, a randomized, double-blind, placebo-controlled trial evaluated the effects of baicalin on the inflammatory profile and lipid levels in patients with coronary artery disease and rheumatoid arthritis. It has been shown, that baicalin (at a dose of 500 mg/day for 12 weeks) have a good safety profile and, compared to placebo, improved total cholesterol levels (2.87 ± 1.23 vs. 3.22 ± 1.07 mmol/L), LDL (1.38 ± 0.41 vs. 1.16 ± 0.32 mmol/L), triglycerides (1.12 ± 0.36 vs. 1.87 ± 0.46 mmol/L), apolipoprotein (1.31 ± 0.41 vs. 1.23 ± 0.29 g/L), hs-CRP (1.64 ± 0.38 vs. 3.9 ± 1.4 mg/dL) and CT-1 (42.9 ± 13.7 vs. 128.4 ± 24.3 ng/mL) [71].

Resveratrol is a natural polyphenol found in over 70 species of plants, including grapes, cranberries, blueberries, and peanuts, but also found in many pharmaceutical formulations. It has been shown, that resveratrol has strong anti-aging, -inflammatory, -apoptotic, -oxidative, -cancerous, -diabetic, hepato- and cardioprotective properties [47]. The antioxidant activity of resveratrol is related to the inhibition of 15-LO and 5-LO in neutrophils, reduction amassment of hydroperoxides in LDL and the decreased in the oxoferryl complex to metmyoglobin, and prevention of LDL modifications promoted by peroxynitrite [12]. Resveratrol reduces posttraumatic axonal degeneration and promotes neurite growth and synaptogenesis in sensory and primary neurons as well as Neuro2a cells [72,73]. In an in vitro study, Tang et al. showed that resveratrol promoted synaptogenesis and neurite growth by activating the sonic hedgehog homolog (Shh) after oxygen–glucose deprivation/reoxygenation (OGD/R) neuronal injury [74]. Neurorestorative properties of resveratrol are also realized through the nuclear factor erythroid 2-related factor 2/heme oxygenase 1 (Nrf2/HO-1) pathway, which results in the inhibition of oxidative stress, neuroinflammation and apoptosis [75]. Importantly, long-term treatment of resveratrol in post-stroke patients (at the dose of 100 and 200 mg, average follow up 12 months, 228 participants, 90 women and 138 men) affected favorably on blood pressure, lipid profile, and body mass index, therefore exhibiting potential adjunctive properties for secondary prevention of stroke [76].

The main active ingredient of turmeric (*Curcuma longa*) is curcumin, which has strong anti-lipidemic, -inflammatory and -aggregating properties, as well as it is an epigenetic modulator and neuroprotective agent [77]. Neurorestorative properties of curcumin in I/R injury are well documented [78,79,80]. Zhou et al. showed that curcumin treatment promoted neuronal viability and inhibited apoptosis. In in vitro studies (isolated neonatal neurons), curcumin reduced the expression of IL-6, Wnt5a, TNFα, the level of JNK1 phosphorylation, and the NFκB nuclear translocation [78]. In turn, Li et al. observed that curcumin reduces brain edema, disruption of the BBB, and infarct volume in I/R injury through anti-inflammatory, -oxidative and -apoptotic activities. Curcumin caused upregulation of Nrf2 expression, decreased expression of NFκB, as well as MMP9, intercellular adhesion molecule 1 (ICAM1), and caspase 3 expression [79,80].

### 2.3. PUFA

In turn, polyunsaturated fatty acids (PUFAs) are part of the phospholipids of cell membranes, which influence the proper functioning and growth of tissues, and are essential modulators of many physiological processes. Importantly, the brain is the second organ, after adipose tissue, with the highest concentration of omega-3 acids (n3-PUFA), thus these acids are necessary for the proper development and functioning of the nervous system. One of the most biologically active n3-PUFAs is docosahexaenoic acid (DHA, 22:6(n-3) in a combined fatty acids nomenclature), which is a component of the phospholipids of neuronal membranes, in particular phosphatidylethanolamine (PE) and phosphatidylserine (PS). It has been shown that a diet low in n3-PUFA promotes synaptic dysfunction and neuronal changes [81]. On the other hand, Saber et al. in prospective study (3675 participants, three cohort, aged > 60, average follow up 11.2 years (Cardiovascular Health Study) and 8.3 years (Nurses’ Health Study and Health Professionals Follow-Up Study) showed that high DHA intake was associated with a lower total stroke risk (HR, 0.80; 95% CI, 0.64–1.00) as well as with decreased risk of atherothrombotic stroke (HR, 0.53; 95% CI, 0.34–0.83) [82]. Moreover, Jiang et al. found that the consumption of n3-PUFa contained in fish oil, including DHA, alleviated post-stroke brain injury and reduced sensorimotor disorders [83]. The antiapoptotic activity of DHA after I/R injury is related with the promotion of translocation and PIP3-depended phosphorylation of Akt and activation of GSK-3β, as well as with the induction of signaling pathways responsible for neuronal survival: protein kinase C (PKC) and Raf-1 kinase [84,85]. The neuroprotective effect of DHA is also based on the activation of antioxidant mechanisms and modulation of neuroinflammation. Experimental stroke animal models revealed that DHA treatment reduced infraction volume, edema, and improved neurobehavior [86,87]. Chang et al. observed in a rat model of permanent cerebral ischemia, that DHA supplementation reduced edema, BBB disruption, infarct volume, and behavioral disturbances by promoting immunosuppression: decreased activation of macrophages/microglia and peripheral leukocytes, as well as the expression of proinflammatory cytokines. Moreover, DHA phosphorylated JNK, c-Jun, activated activator protein 1 (AP-1), and increased the expression of Nrf2 and HO-1 [88].Another n3-PUFA, eicosapentaenoic acid (EPA 20:5(n-3)) in interaction with immune and endocannabinoid system promotes brain cell repair [89]. In the study, EPA is shown to proliferate neural stem cells (NSC) what is associated with enhancing levels of the endocannabinoid 2-arachidonylglycerol (2-AG) and p-p38 MAPK. Contrary, DHA increased proliferation but without effect on p-p38 MAPK. An additional experiment using mice with NSCells deficient in IL-1β, was conducted where EPA lowered proliferation and p-p38 MAPK levels while DHA enhanced proliferation without effect on p-p38 MAPK. That suggest key role of IL-1β signaling and divergent pathway in case of DHA. That phenomenon of different signaling pathways in interaction with endocannabinoid system promises therapeutic functions of interacting DHA and EPA. In consequence supplementing diet with only DHA, what is the case for synthetic product, may be lower or deficient comparing to applying mixture of both n3-acids. Interacts with endocannabinoid system is not reported for α-Linoleic acid (ALA, 18:3(n-3))) what suggest that vegan, and poor in fish diets should be supplemented either with synthetic DHA and EPA sources or extracts from very few non-animal species containing EPA [90].

### 2.4. Macroelements

It has been suggested that high potassium levels may reduce the risk of stroke [91,92]. The protective effect of K^+^ is mainly due to its hypotensive effect, however, as well as is related to its preventive activity on the reduction of free radical production, smooth muscle proliferation, and inhibition of macrophage adhesion to the vascular wall [93]. Increasing the daily intake of K^+^ by 10 mmol is associated with a 40% reduction in the relative risk of stroke mortality. The effect of potassium was independent of other factors: magnesium, calcium, alcohol and calorie intake, dietary fat, protein, and fiber content [92]. Moreover, it has been observed that both the consumption of potassium supplements and the enrichment of the diet with foods containing high amounts of this element reduce the stroke risk [91]. Thus, it is recommended to use a diet rich in potassium or its supplementation, however, there are no studies concluding that serum concentration of potassium optimally reduces the risk of stroke. Pan et al. investigated the effect of enriching salt with potassium and magnesium in the diet of post-stroke patients (291 participants, aged 64.5 ± 9.9, average follow up 6 months) on the clinical parameters of disability in comparison to salt enriched with K^+^ and salt without enrichment (NaCl only). They demonstrated that the use of K^+^/Mg^2+^ salt (47.6 mmol NaCl, 44.9 mmol) KCl and 3.8 mmol MgSO_4_ · 7H_2_O) was associated with an increase in the enhancement of good clinical results (OR 2.25, 95% CI 1.09–4.67) compared to salt without enrichment. Importantly, K^+^ salt (55.6 mmol NaCl and 43.6 mmol KCl) increased the improvement of neurological status by 58%, but it was not statistically significant (OR 1.58, 95% CI 0.77–3.22) [94]. In turn, the neuroprotective activity of magnesium is related to augmentation of regional blood flow to ischemic regions, non-competitive inhibition of glutamate and voltage-sensitive calcium, enhancement of adenosine action, inhibition of glutamate release, increased regeneration of cellular energy metabolism following ischemia [95]. Studies in animal models have shown that magnesium supplementation reduces infarct volume and enhances neurological outcomes [96].

As previously mentioned, the protective effect of the diet is related to the established nutritional regimen, while the data on the protective effect of individual micronutrients and vitamins are often divergent. In addition, restrictions on supplementation of various compounds require some caution, generally playing a significant role in post-stroke recovery. Future research should deepen our understanding of the neuroprotective mechanisms of these compounds and explore how supplementation can be effectively used to treat stroke.

### 2.5. Endogenous Substance

An endogenous substance with a potential neuroprotective effect is the neurohormone secreted by the pineal gland—melatonin (Mel). Mel is an antioxidant lipophilic indoleamine that plays a key role in the modulation of the circadian rhythm by regulating sleep and wakefulness and penetrates the BBB [97]. Mel’s antioxidant effect is achieved by scavenging oxygen and nitrogen free radicals by its metabolites, as well as by activating SOD and GPx and increasing the efficiency of the respiratory chain [98,99]. Pei et al. showed that intraperitoneal pretreatment of Mel (5 or 15 mg/kg) reduced the infarct volume in rats caused by middle cerebral artery occlusion and reperfusion (MCAO/R) [100]. Moreover, other studies in animal models have shown that Mel improved behavioral deficits and reduced damage to brain tissue [101,102], as well as reduced oxidative stress, improved behavioral and cognitive performance, and enhanced neuronal viability [103,104,105]. Mel’s neuroprotective effects are related to its MT1 and MT2 receptors, which are present throughout the brain. It has been shown that Mel promotes neurogenesis through the MT2 receptor and enhanced the neurogenic ability of mesenchymal stem cells [106]. Importantly, in animal models of ischemia, a reduction in the number of MT1 receptors was observed, which led to an increase cell death in cerebral cortex, while treatment with Mel enhanced cell survival by upregulation of MT1 receptors [107]. The involvement of MT2 in neuroprotection is confirmed by the studies of Tang et al. They showed that MT2 activation improves cognitive disorders through the cAMP-C/EBPα/miR-125b/GluN2A pathway in an AD mouse model [108]. In contrast, Zheng et al. reported that treatment with ramelteon, an MT1 and MT2 agonist, reduced functional deficits in mice with both acute and chronic cerebral ischemia. Moreover, ramelteon reduced autophagy in the peri-infarct cortex inhibited by the AMPK/mTOR pathway [109]. Mel may be an effective treatment for post-stroke dementia, where oxidative stress is a major contributing factor. Muhammad et al. noted that Mel promoted neuronal survival and proliferation, increased endogenous antioxidant levels via the Akt/ERK/CREB pathway in an AD mouse model. In addition, Mel reduced apoptosis, memory loss, neurodegeneration, and neuroinflammation [110]. Melatonin also promoted hippocampal neurogenesis by enhancing CREB phosphorylation and increasing BDNF levels [111]. Overall, melatonin may be a promising therapeutic agent in the treatment of stroke, in particular by enhancing cognition function through neuroprotective, anti-inflammatory, antioxidant and antioxidant effects.

γ-Aminobutyric acid (GABA), serves as main cortex inhibitory neurotransmitter, responsible for the inhibitory action on the stretch receptor system and thus is essential in providing balance between excitation and inhibition of neurons [112]. GABA regulates neuronal excitability through binding to specific membrane proteins, what results in opening of an ion channel. The entry of a chloride ion through the ion channel leads to hyperpolarization of the recipient cell, what consequently prevents transmission of nerve impulses to other cells. Some psychiatric disorders are correlated with GABAergic abnormalities [113,114]. GABA is synthesized in a human body from glutamate through alpha-decarboxylation mediated by glutamic acid decarboxylase (GAD) and is consequently metabolized to succinate in a series of GABA-transaminase (GABA-T) and succinic semialdehyde dehydrogenase (SSADH) mediated reactions [115]. GABA is contained in certain food as well as delivered in with certain food products, in particular spinach and sprouted cereals and brown rice germ. Additionally, certain gut bacteria such as Lactobacillus and Bifidobacterium strains are able to synthesize GABA Barret [116].

While research supports or proves association of GABA deficit with the conditions described above, question whether supplementing GABA plays any important role in treating the disorders. The main argument is that GABA does not cross the BBB [117] and the short half-life time of GABA suggests it may decompose prior to reaching from the G-I tract to brain neural cells [118]. On the other side, a number of research reports positive effect of GABA supplementation on such functions as temporal attention, reducing acrophobia, lessening psychological fatigue after completion of the task [119,120,121].

GABA can induce neurons hyperpolarization through presynaptic G-protein coupled receptors (GABA_B_) and anion channels (GABA_A_) [122], which reduces the depolarization that initiates the biochemical ischemic cascade [123]. Moreover, it has been shown that under ischemic conditions GABA deficiency is not observed, but only a decrease in the affinity of their receptors [124]. GABA activation suppresses acidosis and respiratory rate, as well as preserving glucose, leading to improved local cerebral blood flow [125,126]. Another neuroprotective action of GABA receptor agonists in acute stroke is the induction of hypothermia [127]. The results of in vivo preclinical studies using GABA agonists are very promising. Clomethiazole, a GABA agonist, has been shown to reduce stroke volume by 58% and 32% in both a rat ischemic model and in marmoset studies, respectively [128,129]. However, phase III clinical trials did not bring the expected results. In a meta-analysis involving five clinical trials (3838 participants), Lin et al. reported that the use of GABA agonists, chlormethiazole and diazepam, compared to placebo, showed no significant differences in the risk of death and patient dependency after 3 months (RR 1.03, 95% CI 0.96–1.11 and RR, 95% CI 0.94 0.82–1.07, respectively). Importantly, the use of chlormethiazole was associated with the occurrence of rhinitis (RR 4.75, 95% CI 2.67–8.46) and drowsiness (RR 4.56, 95% CI 3.50–5.95) [130]. Considering all of the above studies, it should be noted that GABA agonists are interesting agents in post-stroke supplementation, nevertheless further, well-designed clinical trials involving a large number of patients are necessary.

### 2.6. Other Bioactive Compounds

One of the food ingredients that has a significant impact on health is dietary fiber. Based on the definition proposed by the European Food Safety Authority (EFSA) and the Codex Alimentarius, the European Commission has published an official definition of dietary fiber. Therefore, dietary fiber is polymers consisting of more than three monomers, which are not digested and absorbed in the human small intestine. These include: naturally occurring carbohydrate polymers, non-digestible carbohydrates obtained by modification and non-digestible synthetic carbohydrates [131]. Physical and chemical properties of dietary fiber: viscosity, the ability to ferment and bind water cause a local and systemic reaction of the body leading to an increase in stool weight, an effect on intestinal peristalsis, and fiber affects metabolism and is a food for the intestinal microflora. The World Health Organization recommends that the daily intake of fiber should be around 20–40 g [132].

In a population study (9677 women, aged 45–83 years), Larsson et al. determined a relationship between dietary fiber intake and the risk of a stroke, the follow-up period was 10.3 years. They showed that high intake of total fiber and fiber from fruits and vegetables is associated with a reduction in the occurrence of stroke (after adjusting for other risk factors, for the highest quintile of total and fruit fiber consumption RR 0.90, 95% CI 0.81–0.99, *p* = 0.003 and RR 0.85, 95% CI 0.77–0.95, *p* < 0.0001, respectively). However, there was no correlation between the risk of stroke and the consumption of cereal fiber (RR 0.94, 95% CI 0.84–1.04, *p* = 0.35) [133]. Similar conclusions were made by Tong et al. In a large cohort study involving 418,329 participants (140,117 men and 278,212 women, aged 52.0 ± 10.1 and 50.4 ± 10.4, respectively) from nine European countries, the follow-up was 12.7 years, it was shown that dietary fiber consumption was associated with a lower risk of ischemic stroke (per 10 g/day, HR 0.77, 95% CI 0.69–0.86, *p* < 0.001) [134]. Confirmation of inverse relationship between dietary fiber intake and risk of stroke is a meta-analysis conducted by Threapleton et al. (per 7 g/day RR 0.93, 95% CI 0.88–0.98, I^2^ = 59%) [135]. Due to frequent disturbances in food intake in post-stroke patients, the addition of powdered dietary fiber to meals could have beneficial effects.

The soluble fiber components are β-glucans, carbohydrate biopolymers that are structural components of plant cell walls (mainly oats and barley), as well as yeasts (including *Saccharomyces cerevisiae*, *Saccharomyces fragilis*, *Candida tropicalis*, *Candida utilis*). β-glucans are strong stimulants and modulators of the immune system, with antiviral, antibacterial and anticancer properties. Moreover, it has been shown that these compounds regulate the body’s carbohydrate and lipid metabolism, lowering the level of triglycerides, cholesterol and glucose, and also have anti-hypertensive effect [136]. Therefore, they positively influence the risk factors of cerebrovascular diseases, thus playing an important role in the primary prevention of stroke. Anticoagulant activity seems to be a promising property of fungal β-glucans in stroke. Mendes et al. demonstrated that (1 → 3) -β-glucan from Botryosphaeria rhodina MAMB-05 dose-dependently prolonged APTT and PT in vitro [137]. Importantly, the anticoagulant activity of β-glucan is similar to heparin, directly inhibiting thrombin and the production of proteases [138].

The active ingredients of *Salvia miltiorrhiza* Bunge, mainly tanshinone I and tanshinone IIA, are widely used in Chinese medicine for the treatment of cardiovascular diseases and ischemic stroke. Tanshinones are lipophilic diterpenoids capable of penetrating the BBB. The main biological activity of these biocomponents is related to the antioxidant and anti-inflammatory activity. Nevertheless, in recent years, growing attention has focused on the neuroprotective effects of these compounds [139]. A serious limitation of the use of tanshinones is low water solubility and exposure to first pass metabolism, thus they are characterized by low oral bioavailability, however, the use of nanocapsules improves their clinical significance [140]. In animal models with induced ischemic stroke, it was shown that intraperitoneal administration of tanshinones at a dose of 4–8 mg/kg inhibited the progression of ischemia by inhibiting neuronal apoptosis [141], reducing oxidative damage to biomolecules [142], and reducing microglia activation [143]. Tanshinones increased the expression of both the gene and the Nrf2 protein, thus enhancing the activity of antioxidant enzymes [142]. Moreover, Wang et al. demonstrated that sodium tanshinone II (10, 20, 40 mg/kg) reduced stroke volume and neurological deficits in MCAO/R mice. The neuroprotective effect was expressed by the inhibition of neuroinflammation (reduction of the number of B lymphocytes, T lymphocytes and macrophages in the ischemic brain), as well as autophagy (decreased up-regulation of LC3-II, Sirt 6 and Beklin-1 proteins) [144].

Iridoids forms a group of monoterpenoids, of the cyclopentanopyrane backbone. Secoiridoids having an open cyclopentane ring display similar properties and are often discussed alongside them. Iridoids are widely present in the plant kingdom [145], in the genera renown in natural medicine such as: Acanthaceae, Rubiaceae, Scrophulariaceae and Valerianaceae, while secoiridoids are reported in Gentianaceae and Oleaceae [146,147,148]. Iridoids are acetals naturally present in a form of glycosides. The best known are geniposide, loganin, acetylbarlerin, deacetylasperulosidic acid, and brasoside (iridoids) and swertiamarine, gentiopicroside, qinjiaoside A, qinjiaoside B, sweroside, and oleuropein (secoiridoids). Iridoids have lipophilic properties what enables them to cross blood–brain barrier. They are reported to have various bio-active, properties in particular the properties of endogenous neurotrophic factors including activity against neurodegenerative diseases such as AD [145,149]. For instance, carvacol improves neurological deficits; reduces cerebral edema and Evans blue leakage; decreases AQP4 mRNA in a dose-dependent manner; reduces AQP4 protein expression in the perihematomal area. Carvacol reduces the oxidative stress in the cerebral cortex; regulates the activities and concentration of SOD, glutathione peroxidase and catalase (MDA level not altered); reduces the levels of soluble Aβ40 and Aβ42 in the cerebral cortex; effects regulated by IDE; improves learning and memory in Morris water maze test. Geniposide decreases the concentrations of cerebral Aβ1-40 and Aβ1-42; up-regulates the protein levels of β-site APP cleaving enzyme (BACE1) and IDE; decrease the protein levels of ADAM10 [150,151]. In a systematic review, Zheng et al. investigated the neuroprotective effects of catapol in an animal model of acute ischemic stroke. From 25 studies (805 animals), they showed that catapol caused a reduction in infarct size (*p* < 0.05). Moreover, the meta-analysis showed that this iridoid, compared to the control, improved neurological functions according to: Zea Longa score (standard mean difference (SMD) −1.14, 95% CI −1.44–−0.85, *p* < 0.00001), Bederson score (SMD −0.84, 95% CI −1.41–−0.27, *p* < 0.01), Adhesive removal test (SMD −1.15, 95% CI −1.69–−0.60, *p* < 0.0001), Bar-grasping test (SMD 1.41, 95% CI 0.66–2.16, *p* < 0.001), and Corner test (SMD −1.72, 95% CI −2.50–−0.94, *p* < 0.0001) [152]. The size of iridoid family makes it difficult to generalize and summarize effects. Additionally, despite the huge research interest, the existing in vivo and in vitro evidences require further investigation to understand fully the neurotropic activity of that family of compounds.

The health-promoting properties of vegetables from the *Brassicaceae* family (including cabbage, Brussels sprouts, broccoli) are mainly related to the presence of biologically active isothiocyanates, which are also available in the form of pharmacological supplements. The best known representative of this group of compounds with potential clinical application is sulforaphane (SFN). SFN is a sulfur-containing phytochemical with anti-inflammatory, antioxidant and chemoprotective properties [153]. Initially, research on the effects of SFN focused on the effectiveness in the prevention and treatment of cancer, but in recent years, the neuroprotective effect of SFN has become growing popular. In vitro studies have shown that SFN, by activating the Nrf2/ARE pathway, has a neuroprotective effect against H_2_O_2_ in relation to cortical neurons, as well as against photo-oxidative damage to retinal pigment epithelial cells [154,155]. In contrast, Zhao et al. showed that systemic administration of SFN 15 min after focal cerebral ischemia in rats caused a reduction in the volume of stroke [156]. In turn, Wu et al. noted that in primary cultures of cortical neurons of Sprague-Dawley rats subjected to OGD, SFN increased cell viability, Bcl-2 expression, and increased caspase 3 levels via the P13/Akt pathway [157]. SFN also has a neuroprotective effect by suppressing the inflammatory response induced by ischemia. SFN in a rat ischemia model reduced brain edema, reduced BBB disruption, and significantly reduced the level of pro-inflammatory cytokines: IL-1β and TNF-α, suppressed the activity and expression of iNOS and COX-2 and NO concentration by inhibiting the NF-κB pathway [158]. The inhibition of neuroinflammation by SFN was confirmed by the studies of Yu et al., which aimed to determine the effect of SFN on the inhibition of NLRP3 inflammasome activation in adult rats with I/R injury. The intraperitoneal administration of sulforaphane (5 and 10 mg/kg) resulted in a significant reduction in the stroke volume as well as improved neurological outcomes compared to the control group. Moreover, SFN suppressed the activation of the NLRP3 inflammasome and the down-regulation of caspase-1, and decreased the expression of pro-inflammatory cytokines: IL-1β and IL-18 [159].

Capsaicin contained in *Capsicum annuum* L. is a well-known activator of nociceptive neurons by enhancing the flow of cations through the membrane. The neuroprotective effect of capsaicin is associated with a reduction in calcium ion influx and inhibition of excitotoxicity, oxidative stress and neuroinflammation, leading to increased survival of neurons [160,161,162]. In in vitro studies, capsaicin treatment of the primary hippocampal neurons of the hypoxia-reoxygenated rats resulted in the inhibition of caspase-3 and the production of ROS by activating the PI3K/Akt pathway leading to reduction of apoptosis and oxidative stress [160]. Moreover, capsaicin decreased hyperlocomotion, memory impairment, and increased the survival of pyramidal cells in the CA1 subfield in gerbils subjected to global ischemia [162]. Huang et al. investigated the effect of direct intra-infarct capsaicin administration in MCAO rats as well as the effect of this compound on glutamate excitotoxicity in cultured cortical neurons. They observed that capsaicin reduced stroke volume and improved motor coordination and behavioral evaluation. In vitro, capsaicin reduced calcium influx and enhanced neuronal survival by down-regulating receptor function and expression, and is dependent on TRPV1 receptors [161].

Glycyrrhizin is the main active ingredient of the *Glycyrrhiza glabra* root and is chemically composed of glycyrrhizic acid and two molecules of glucuronic acid. The biological activity of glycyrrhizin is primarily associated with anti-inflammatory, anti-allergic, hepatoprotective, antitumor and antiviral effects [163]. The anti-inflammatory effect of glycyrrhizin is expressed by inhibition of high mobility group box chromosomal protein 1 (HMGB1) [164]. In the acute phase of stroke, stimulation of the effectors of the innate immune response responsible for the removal of dead cells is observed. Sudden death of neuronal cells at the site of hypoxia and the release of damage-associated molecular patterns (DAMP) constitute the essence of the activation of local immune processes in the brain. One of the pro-inflammatory factors is HMGB1 produced by NFκB [165]. The neuroprotective effect of glycyrrhizin has been confirmed in animal models of ischemia and is related with anti-inflammatory, antioxidant, antiapoptotic and anti-excitotoxic effect. It has been found, that this compound improved locomotor deficits, reduced infarct volume and cerebral edema in treated animals [166,167,168,169,170]. Kim et al. showed that the intravenous injection of glycyrrhizin (10 mg/kg) to MCAO rats was associated with the improvement of neurological deficits and motor impairment, but also with the suppression of activation of proinflammatory cytokines and microglia [168]. In experimental studies, this compound suppressed neuroinflammation through the TLR4/NF-κB, P38 and P-JNK pathways, which was manifested in the inhibition of HMGB1 release from the cerebral cortex, lowering the level of pro-inflammatory cytokines: TNF-α, iNOS, IL-1β and IL-6 as well as the attenuation of IFNγ expression in TCD4 lymphocytes [168,169,171]. Moreover, glycyrrhizin (at a dose of 20 mg/kg for 5 days) reduced cognitive impairment through suppressed long-term potentiation induction, memory enhancement, inhibition of voltage-gated sodium channels (VGSCs) in hippocampal CA1 pyramidal neurons, and reduction of oxidative stress in vascular dementia rat model [171]. To date, no clinical trials have been performed on the treatment of glycyrrhizin in post-stroke patients. However, based on clinical trials of other disease entities for which the therapeutic target is the suppression of inflammation, it may be concluded that glycyrrhetic acid (glycyrrhizin metabolite) is a strong inhibitor of 11 β-hydroxysteroid dehydrogenase, and thus has an effect similar to mineralocorticoids. Side effects of treatment with glycyrrhizin include: hypokalemia, cardiac arrhythmia, metabolic alkalosis, edema and arterial hypertension [172].

## 3. Conclusions

The increase in the incidence of cerebral vascular diseases implies major health and social consequences, thus it is a strong impulse to search for new therapeutic forms all over the world. The use of natural compounds with a low degree of toxicity seems to be an important research direction. The inclusion of supplementation in the standard therapy is aimed at preventing malnutrition and supporting the natural healing processes. Among the compounds of particular importance are vitamins, antioxidant compounds such as flavonoids, resveratrol, polyunsaturated fatty acids, as well as fiber, tanshinones and sulforaphane. The participation of natural compounds in primary prevention of stroke and supporting post-stroke recovery are summarized in Table 1. Despite promising preclinical research, the exact effect on the human body requires further research. The appropriate administration method and doses cannot be clearly stated. Certainly, the essence of stroke prevention is preventing vitamin and mineral deficiencies. Thus, the administration of B vitamins in doses: folic acid—0.5 mg/day; B12—0.5 mg/day, B6—16.5 mg/day, seems to be justified [173]. In addition, treatment of vitamin D deficiency may consist of the oral administration of ergocalciferol at a dose of 50,000 IU/week for 8 weeks, and after reaching the appropriate level—800–1000 IU/day [174]. On the other hand, high intravenous administration of vitamin C in the form of dehydroascorbic acid seems to be clinically significant [175]. Further studies are necessary to determine both the clinical dose and the route of administration, taking into account the pharmacodynamics and pharmacokinetics of the compounds.

## Figures and Tables

**Table 1:** The participation of natural compounds in primary prevention of stroke and supporting post-stroke recovery.

	Natural Compounds	Biological Activity in Central Nervous System	Literature
Primary stroke prevention	B vitamins (B6, B12, folic acid)	Inhibition of atherosclerotic processes by involvement in homocysteine methylation	[25,26,27,46]
	Carotenoids	Reduction of fibroblast growth factor-1 (FGF1)-mediated gliosis of astrocytes by increasing the expression of genes related to cholesterol regulation: Abcg2, Abca1, Hmgcr, and Apoe Reducing the risk of death from stroke	[31,32,33]
	Polyunsaturated fatty acids (PUFAs)	Reduction of lower total stroke risk and decreased risk of atherothrombotic stroke Alleviation of post-stroke brain damage and reduction of sensorimotor disorders	[82,83]
	Potassium	Anti-hypotensive effect Reduction of free radical production, smooth muscle proliferation, and inhibition of macrophage adhesion to the vascular wall Reduction of the stroke risk	[91,93]
	Dietary fiber	Reduction of stroke risk	[133,134,135]
	β-glucans	Strong stimulants and modulators of the immune system Anti-viral, antibacterial, anti-hypertensive and anticancer properties Regulation of the body’s carbohydrate and lipid metabolism, lowering the level of triglycerides, cholesterol and glucose	[136]
	Vitamin C	Antioxidative and anti-inflammatory properties Dose-dependently reduction of infract volume, mortality, edema, and neurological disorders Improvement of neurological outcomes as well as blood flow	
Neuroprotection	Vitamin D	Improvement of cerebral blood flow, reduction of blood pressure, and vasodilation by increasing the activity of nitric oxide synthase (NOS) Enhancement of the expression of neurotrophic factors (vascular endothelial growth factor—VEGF, stromal cell-derived factor 1α—SDF1α, and insulin-like growth factor 1—IGF-1) Reduction of neuronal degeneration Prevention of blood–brain barrier (BBB) disturbance by inhibiting oxidative stress and regulation of tight-junction protein occludin and claudin-5 expression	[16,19,22]
	Flavonoid-rich food (FRF)	Improving cognitive function, regardless of age and medical history Reducing neuronal apoptosis and scavenging free radicals, as well as inhibiting neuroinflammation Reduction of proinflammatory biomarker expression (IL-1β, IL-6, IL-4, TNF-α, inducible nitric oxide synthase—iNOS, nuclear factor kap-pa-light-chain-enhancer of activated B cells NFκB, matrix metalloproteinase-9—MMP-9, and cyclooxygenase-2—COX-2) Decrease in the level of protein kinase RNA-like kinase endoplasmic reticulum (p-ERK), N-terminal c-jun kinase (p-JNK), and members of mitogen-activated protein kinase (MAPK) pathway The molecular neuroprotective mechanism associated with the activation of the cAMP response element-binding protein (CREB)/brain-derived neurotrophic factor (BDNF)/tropomyosin-related kinase B receptor (TrkB)/phosphoinositide 3-kinase (PI3K)/protein kinase B (Akt) and/or ERK 1/2 pathways	[47,48,49,50,51,52]
	Quercetin	Anti-oxidant, -inflammatory, -platelet, -atherosclerotic, -obesity, -hypercholesterolemic, -cancer, and -allergic properties inhibiting cellular toxicity Reduction of both systolic diastolic blood pressure	[53,59]
	Baicalin	Enhancement of cognitive, behavioral and motor functions Improvement of neurological deficit Decrease in the infarct volume Enhancement of synaptic plasticity	[65,66,67,68,69,176]
	Epigallocatechin gallate	The activation of CREB/BDNF/TrkB-PI3K/Akt signaling Increases in Akt, phospho-Akt, mTORc1 and phospho-glycogen synthase kinase 3 (pGSK3b), as well as growth in BDNF and TrkB expression Decreases in neurological deficits Reduction of the level of brain injury and oxidative stress biomarkers Inhibition of neuronal apoptosis Promoting neuron survival	[61,63]
	Resveratrol	Anti-aging, -inflammatory, -apoptotic, -oxidative, -cancerous, -diabetic, hepato- and cardioprotective properties Reducing post-traumatic axonal degeneration and promoting neurite growth and synaptogenesis by activating the sonic hedgehog homolog (Shh) after oxygen–glucose depriva-tion/reoxygenation (OGD/R) neuronal injury The inhibition of oxidative stress, neuroinflammation and apoptosis Beneficial effect on blood pressure, lipid profile and body mass index in post-stroke patients	[72,73,74,75,76]
	Curcumin	Anti-lipidemic, -inflammatory and -aggregating properties Epigenetic modulator and neuroprotective agent Promoting neuronal viability Inhibition of apoptosis Reduction of the expression of IL-6, Wnt5a, TNFα, the level of JNK1 phosphorylation, and the NFκB nu-clear translocation Reduction of brain edema, disruption of the BBB, and infarct volume Upregulation of Nrf2 expression Decrease in expression of NFκB, as well as MMP9, intercellular adhesion molecule 1 (ICAM1), and caspase 3 expression	[77,78,79,80]
	Docosahexaenoic acid (DHA)	Promotion of translocation and PIP3-depended phosphorylation of Akt and activation of GSK-3β The induction of signaling pathways responsible for neuronal survival: protein kinase C (PKC) and Raf-1 kinase The activation of antioxidant mechanisms and modulation of neuroinflammation Reduction of infraction volume, edema, BBB disruption, infarct volume, and improved neurobehavior Promoting immunosuppression: decreased activation of macrophag-es/microglia and peripheral leukocytes, as well as the expression of proinflammatory cytokines Phosphorylation of JNK, c-Jun, activated activator protein 1 (AP-1), and increased the expression of Nrf2 and HO-1	[84,85,86,87,88]
	Eicosapentaenoic acid (EPA)	Interaction with immune and endocannabinoid system promotes neurorepair Augmenting proliferation of neural stem cells (NSC) what is associated with enhancing levels of the endocannabinoid 2-arachidonylglycerol (2-AG) and p-p38 MAPK	[89,90]
	Magnesium	Augmentation of regional blood flow to ischemic regions Non-competitive inhibition of glutamate and voltage-sensitive calcium Enhancement of adenosine actionInhibition of glutamate release Increased regeneration of cellular energy metabolism Reduction of infarct volume and enhancing neurological outcomes	[94,95]
	Melatonin	Reducing the infarct volumeImprovement of behavioral deficits and reduction of damage to brain tissue Inhibition of oxidative stress Enhancement of neuronal viability Promoting neuronal survival and proliferation Increase in endogenous antioxidant levels via the Akt/ERK/CREB pathway Inhibition of apoptosis, memory loss, neurodegeneration, and neuroinflammation Promoting hippocampal neurogenesis by enhancing CREB phosphorylation and increasing BDNF levels	[101,102,103,104,105,110]
	γ-Aminobutyric acid (GABA)	Positive effect on such functions as temporal attention, reducing acrophobia, lessening psychological fatigue after completion of the task	[119,120,121]
	Tanshinones	Inhibition of the ischemia progression by reducing neuronal apoptosis Reducing oxidative damage and microglia activation Increase in the expression of both the gene and the Nrf2 protein Enhancing the activity of antioxidant enzymes Inhibition of neuroinflammation (reduction of the number of B lymphocytes, T lymphocytes and macrophages in the ischemic brain), as well as autophagy (decreased up-regulation of LC3-II, Sirt 6 and Beklin-1 proteins)	[141,142,143,144]
	Carvacrol	Improvement of neurological deficits Reducing cerebral edema and Evans blue leakage Decrease in AQP4 mRNA in a dose-dependent manner Reduction of AQP4 protein expression in the perihematomal area Reducing the oxidative stress in the cerebral cortexRegulation of the activities and concentration of SOD, glutathione peroxidase and catalase Reducing the levels of soluble Aβ40 and Aβ42 in the cerebral cortex Improvement of learning and memory Up-regulation of the protein levels of β-site APP cleaving enzyme (BACE1) and IDE Decrease in the protein levels of ADAM10	[150,151]
	Glycyrrhizin	Anti-inflammatory, antioxidative, antiapoptotic and anti-excitotoxic properties Improvement of locomotor deficits Reduction of infarct volume and cerebral edema	[163,166,167,168,169]
	Sulforaphane	Anti-inflammatory, antioxidant and chemoprotective properties Increase in cell viability, Bcl-2 expression, and increased caspase 3 levels via the P13/Akt pathway Suppressing the inflammatory response induced by ischemia Reducing brain edema, BBB disruption, and the level of pro-inflammatory cytokines: IL-1β and TNF-α Suppressing the activity and expression of iNOS and COX-2 and NO concentration by inhibiting the NF-κB pathway Reduction of the stroke volume and improvement of neurological outcomes Suppressing the activation of the NLRP3 inflammasome and the down-regulation of caspase-1, and decrease in the expression of pro-inflammatory cytokines: IL-1β and IL-18	[153,157,158,159]
	Capsaicin	Reduction of calcium ion influx and inhibition of excitotoxicity, oxidative stress and neuroinflammation, leading to increased survival of neurons Inhibition of caspase-3 and the production of ROS by activating the PI3K/Akt pathway leading to reduction of apoptosis and oxidative stress Decreased in hyperlocomotion, memory impairment Increase in the survival of pyramidal cells in the CA1 subfield Reduction of stroke volume and improvement of motor coordination and behavioral evaluation	[160,161,162]
